# Irradiation-Hardening Model of TiZrHfNbMo_0.1_ Refractory High-Entropy Alloys

**DOI:** 10.3390/e26040340

**Published:** 2024-04-17

**Authors:** Yujun Fan, Xuejiao Wang, Yangyang Li, Aidong Lan, Junwei Qiao

**Affiliations:** 1 College of Materials Science and Engineering, Taiyuan University of Technology, Taiyuan 030024, China; fanyujun1019@126.com (Y.F.); lanaidong@tyut.edu.cn (A.L.); 2 Key Laboratory of Interface Science and Engineering in Advanced Materials, Ministry of Education, Taiyuan University of Technology, Taiyuan 030024, China; wangxuejiao@tyut.edu.cn; 3 Huaxin Gas Group Co., Ltd., Taiyuan 030000, China; 13852106610@163.com

**Keywords:** high-entropy alloys, mechanical properties, ion irradiation, indentation size effect, plastic zone

## Abstract

In order to find more excellent structural materials resistant to radiation damage, high-entropy alloys (HEAs) have been developed due to their characteristics of limited point defect diffusion such as lattice distortion and slow diffusion. Specially, refractory high-entropy alloys (RHEAs) that can adapt to a high-temperature environment are badly needed. In this study, ${TiZrHfNbMo}_{0.1}$ RHEAs are selected for irradiation and nanoindentation experiments. We combined the mechanistic model for the depth-dependent hardness of ion-irradiated metals and the introduction of the scale factor f to modify the irradiation-hardening model in order to better describe the nanoindentation indentation process in the irradiated layer. Finally, it can be found that, with the increase in irradiation dose, a more serious lattice distortion caused by a higher defect density limits the expansion of the plastic zone.

## 1. Introduction

With the continuous development of the economy and society, the large demand for energy and environmental protection should achieve a relative balance. Therefore, the development of clean energy has become the theme. Among many clean energy sources, nuclear energy has great application prospects. The overwhelming majority of structural materials play a pivotal role in nuclear reactors. In general, the conventional materials for nuclear reactors include various ferritic/martensitic steels [1], austenitic stainless steel [2], zirconium alloys [3], ceramics, composite materials, etc. In future nuclear systems, their ability to withstand high radiation doses and harsh environments has certain limitations. Therefore, it is necessary to find excellent nuclear structural materials that are resistant to radiation damage [4].

Yeh et al. [5] proposed high-entropy alloys (HEAs) in 2004, and they have attracted considerable attention as a potential material. According to the composition definition, HEAs contain four or more elements in equal or near-equal proportions, with an element concentration between 5% and 35%. Due to the highly disordered solid solution matrix and the inherent strain lattice, the solute diffusion of HEAs is slow [6]. In addition, due to the existence of a size difference and modulus difference for the constituent elements, HEAs usually have a large lattice distortion [7,8]. These all hinder the formation and migration of point defects and defect clusters generated by irradiation, which is the reason for the excellent radiation damage resistance of HEAs [9]. Among HEAs, refractory high-entropy alloys (RHEAs) with excellent high-temperature mechanical properties are more suitable for high-temperature environments (300 °C–800 °C) for irradiation. Up until now, there are many studies on the irradiation behavior of face-centered cubic (FCC) structured HEAs, and only a few studies focus on the irradiation behavior of body-centered cubic (BCC) structured RHEAs [10].

The difficulty in the application of RHEAs is their room-temperature brittleness [11]. The TiZrHfNbTa alloy [12] is one of the few alloys with a macroscopic room-temperature tensile plasticity, but its high-temperature performance is poor. Compared with the TiZrHfNbTa alloy, the TiZrHfNbMo alloy [13] has a higher strength at high temperatures, but the plasticity of the TiZrHfNbMo alloy is poor at room temperature. By adjusting the content of the Mo element, the TiZrHfNbMo_0.1_ alloy has a balanced room-temperature tensile strength and plasticity. Its performance in the irradiation environment is studied in this study.

Compared with neutron irradiation, the ion irradiation used in the experiment only acts on the surface of the sample. But it is more convenient, safer, and faster. As an effective and convenient method, nanoindentation has been widely used to analyze the mechanical behavior of the thin film [14], fiber [15] and thin surface layer of ion-irradiated metallic materials [16,17]. These studies [18] reflect the indentation size effect similar to that of unirradiated materials, that is; the hardness increases as the indentation depth decreases, especially in the submicron depth range. In contrast, the Nix–Gao model [19] cannot capture the hardening caused by irradiation-induced defects and the uneven distribution of defects, nor can it reflect the soft matrix effect. Xiao et al. [20] constructed a complete model for the hardness variation with the depth to solve these problems, but there may be some differences in the application for HEAs.

In this study, the irradiation damage experiments and nanoindentation for ${TiZrHfNbMo}_{0.1}$ RHEAs are carried out. Combined with the irradiation-hardening model, the scale factor f is introduced to reflect the change of the plastic zone radius under indentation. Compared with the samples before irradiation, the plastic zone and scale factor f of the samples with a different dpa change continuously. Then, through the data analysis of the irradiation-hardening model, combined with the irradiation damage evolution in previous studies, the change of the plastic zone is reasonably explained.

## 2. Methods and Experimental Procedures

### 2.1. Sample Preparation

RHEAs with a nominal composition of ${TiZrHfNbMo}_{0.1}$ were prepared by arc melting of pure metal mixture (99.9 wt. %) in high-purity Ar atmosphere. The ingot was turned over and remelted at least six times to promote chemical homogeneity, and then suction-cast into a mold with a thickness of 6 mm. The as-cast ingot was cold-rolled to a thickness of 80%, and then the plate was cut into a dog-bone-like tensile sample by wire cutting. After that, the tensile sample was homogenized at 1100 °C for 1 h/6 h/12 h, and then quenched in water. During the annealing process, all samples were sealed in a quartz tube filled with high-purity argon to prevent oxidation. The Instron 5969 testing machine was used for quasi-static uniaxial tensile tests at a strain rate of 10^−3^/s at room temperature. To ensure repeatability, at least three tensile samples were produced under each tensile condition. Prior to irradiation experiments, samples processed from uniform flakes were first ground with sandpaper, then polished with the diamond plaster, and finally electropolished with the solution of ${6\%HClO}_{4}\;$+ ${35\%C_{4}H}_{10}$O + ${59\%CH}_{3}$OH to remove any work-hardened surface layers. Co-Kα radiation (*k* = 0.154 nm) was measured by XRD of different samples using PANalytical diffractometer. The scanning range was from 20° to 100°. The initial microstructure of the polished sample was characterized by Phenom XL scanning electron microscope.

### 2.2. Ion Irradiation

The nuclear transmutation reaction induced by 14-MeV neutron flux could produce a large number of He atoms, indicating that it has a negative impact on the mechanical properties of materials, such as hardening and embrittlement [16]. Studying the effect of He ion irradiation on the mechanical properties of RHEAs was of great significance for the preliminary evaluation of irradiation performance.

**Figure 1:**
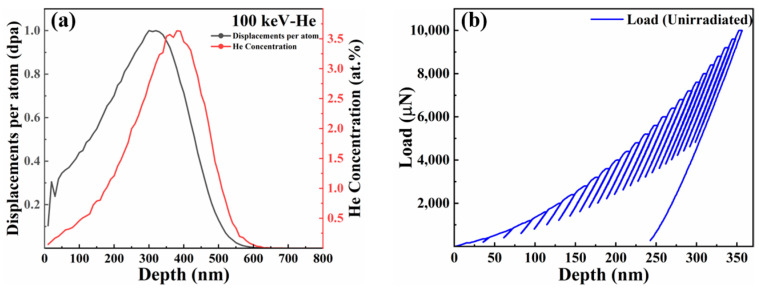
(**a**) The depth-dependent dislocation damage (dpa) and ${He}^{+}$ distribution obtained by SRIM simulation of the ${TiZrHfNbMo}_{0.1}$ alloy, and (**b**) variation curve of load with the depth in nanoindentation (cyclic loading).

In the Kinchin–Pease model, the stopping and range of ions in matter (SRIM) [21] was used to simulate the displacement and injected He ion profiles at highest irradiation flux. It is assumed that the threshold displacement energies of Ti, Zr, Nb, Hf, and Mo elements are 30, 40, 78, 90, and 60 eV, respectively, which is consistent with the literature [22]. Figure 1a exhibits the SRIM prediction results of displacement and implanted He ions with the depth distribution in ${TiZrHfNbMo}_{0.1}$ alloy. The formula is as follows [16]:

(1) $$dpa=\left(\frac{vacancies}{ions\times Ang.}\right)\times 10^{8}\times \frac{\Phi }{N}$$

where $\left(\frac{vacancies}{ions\times Ang.}\right)$ is the maximum value of the vacant file output based on SRIM software (http://www.srim.org/). Φ is the ion dose, the unit is $ions/{cm}^{2}$, and N is the atomic density, and the unit is $atom/{cm}^{2}$.

According to the simulation results of SRIM, assuming that the peak damage is 1 dpa, the irradiation flux required by the material is 4.7 × 10^16^$\;ions/{cm}^{2}$. Since a certain amount of heat was generated during ion implantation, the samples were prepared by 100 kev He ion irradiation at about 100 °C using CI-S200 ion implantation machine at Taiyuan University of Technology, and the flux reached 4.7 × 10^14^$\;ions/{cm}^{2}$ (0.01 dpa), 4.7 × 10^15^$\;ions/{cm}^{2}$ (0.1 dpa), and 4.7 × 10^16^$\;ions/{cm}^{2}$ (1 dpa), respectively.

### 2.3. Nanoindentation

The nanoindentation test was performed using a Hysitron TI Premier nanoindentation machine (Bruker, Minneapolis, MN, USA) with a Berkovich tip (radius ~162 nm) at room temperature. Irradiated and unirradiated materials for hardness measurement are TiZrHfNbMo_0.1_ RHEA after 1100 °C/1 h heat treatment. The most straightforward technique for mounting samples is to adhere the sample to a steel SPM puck with a cyanoacrylate-based adhesive (super glue). Try to ensure the level of the sample and the stage. Before the experiment, the tip radius was calibrated on the reference fused silica sample. In the load control mode, a cyclic loading was performed on each test point at a fixed loading rate of 2 mN/s and a maximum load of 10 mN. In order to make the data repeatable enough, a cyclic loading nanoindentation test of 25 experimental points was performed on each sample, as shown in Figure 1b. The grid indentation was used, and the horizontal and vertical intervals between continuous indentations were 20 μm to reduce the influence of indentations on each other. These experimental points were carried out on the same equiaxed grain after homogenization, so as to weaken the influence of grain boundary on nanoindentation [23]. The experimental hardness was determined by analyzing the load–displacement (P-H) curve using the Oliver and Pharr methods [24]. After that, the hardness data of different depths were output, and the hardness data points changing with depth were generated.

## 3. Results and Discussion

### 3.1. Microstructure and Mechanical Properties of the Alloy before Irradiation

**Figure 2:**
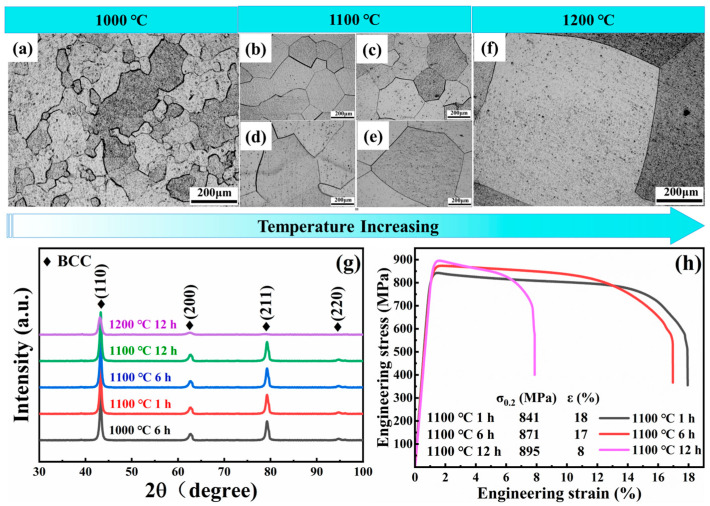
The metallographic diagram ((**a**) 1000 °C/6 h, (**b**) 1100 °C/1 h, (**c**) 1100 °C/6 h, (**d**) 1100 °C/12 h, (**e**) 1100 °C/24 h, and (**f**) 1200 °C/12 h) and corresponding XRD diagram (**g**) of the alloy heat treated at different temperatures and time, and the stress–strain curve (**h**) at 1100 °C for different time.

After the alloy is rolled by 80%, the grains are broken, and then the alloy is recrystallized after heat treatment at the temperatures of 1000 °C/1100 °C/1200 °C and the corresponding time of 1 h/6 h/12 h. The metallographic diagram and XRD pattern of the ${TiZrHfNbMo}_{0.1}$ alloy after heat treatment at different temperatures and time are shown in Figure 2a–g. It can be seen from Figure 2a that the recrystallized grains at 1000 °C/6 h are not uniform. The recrystallized grains with the time of 6 h and 12 h are larger at 1100 °C. The recrystallization grain size (~130 μm) at 1100 °C/1 h is more suitable and the distribution is more uniform. The XRD pattern shows that the alloys are in the single-phase BCC phase, indicating that the alloy has excellent phase stability at high temperatures [13,25]. The existence of single-phase and uniform grains paves the way for the smooth progress of the following nanoindentation. Figure 2h is the quasi-static tensile test of the alloy heat treated at 1100 °C for 1 h/6 h/12 h. The experimental results display that the tensile strength and plasticity of the alloy at 1100 °C for 1 h and 6 h are outstanding. In the case of similar strength, the alloy with better plasticity after heat treatment at 1100 °C for 1 h is selected to carry out subsequent irradiation experiments. The inverse Hall–Petch relation in Figure 2h—that is, the smaller the grain, the lower the strength—may be due to the rotation of grains and the migration of grain boundaries [26].

**Figure 3:**
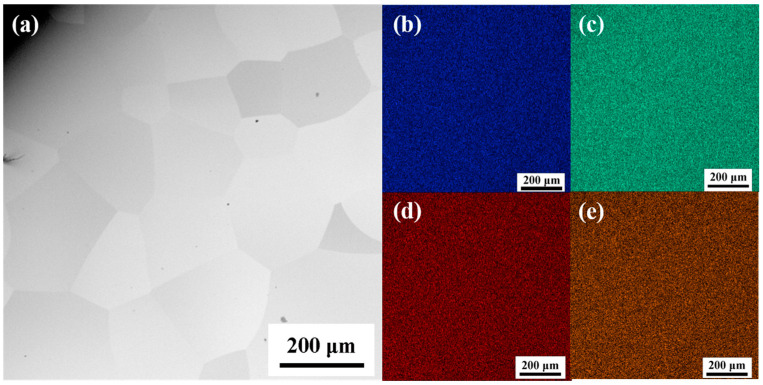
(**a**) The SEM image of the ${TiZrHfNbMo}_{0.1}\;$alloy with equiaxed grain microstructure after heat treatment at 1100 °C for 1 h; and (**b**–**e**) corresponding EDS maps of Nb, Hf, Zr, and Ti, respectively.

In order to reduce the influence of other factors on the subsequent studies, the ${TiZrHfNbMo}_{0.1}$ alloy was electropolished after homogenization at 1100 °C for 1 h to remove the surface stress [27], as shown in Figure 3. The SEM-EDS diagram in Figure 3 exhibits the uniform distribution of the elements of Ti, Zr, Hf, Nb, and Mo, and no additional precipitated phases are generated. Among them, the Mo element cannot be displayed in the figure due to its low content.

### 3.2. Nanoindentation of the Alloy before and after Irradiation

**Figure 4:**
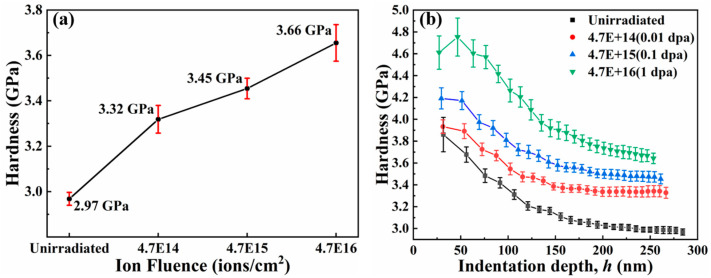
(**a**) The indentation hardness of the ${TiZrHfNbMo}_{0.1}\;$alloy after heat treatment at different irradiation doses, and (**b**) the indentation depth profiles of the nano-hardness measurements of the ${TiZrHfNbMo}_{0.1}\;$alloy at different irradiation doses after heat treatment.

Figure 4 presents the hardness change of the ${TiZrHfNbMo}_{0.1}$ alloy under different irradiation doses and the hardness change with the depth. As the irradiation dose of the sample raises, the hardness values become larger and larger, from the average hardness at the beginning (~2.97 GPa) to the average hardness at the maximum dose (~ 3.66 GPa). The hardening rate (~23.2%) of the alloy is known under 1 dpa irradiation, as shown in Figure 4a and Table 1. Such a hardening rate is terrific in radiation-resistant alloys. In Figure 4b, the material as a whole presents an indentation size effect with increasing depth and decreasing hardness [28,29]. At the early stage of indentation, due to the influence of the surface, the inverse indentation size effect will appear as the indentation depth becomes deeper [30]. Under a fixed load, the maximum depth of the downward pressure of the nanoindentation indenter decreases continuously. This is due to the continuous raising of the irradiation dose, which leads to the increase in the irradiation defects of the sample, and the dislocation is difficult to move by pinning, which makes the hardness increase continuously [28,31]. Therefore, under the same load, the greater the irradiation dose, the shallower the indentation depth in the sample.

#### 3.2.1. Nanoindentation before Irradiation

**Figure 5:**
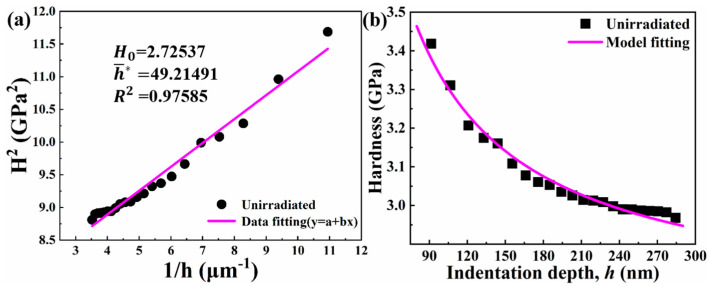
(**a**) The corresponding points and fitting lines of $H^{2}$ vs. 1/h without irradiation, and (**b**) corresponding points and fitting curves of the hardness changing with the depth under unirradiated condition after heat treatment.

Before irradiation, there are no defects caused by irradiation in the alloy. In the irradiation-hardening model proposed by Xiao et al. [20], the model is similar to the Nix–Gao model without considering the irradiation defect density. In addition, based on the assumption that the number of dislocations contained in the effective plastic zone under the contact zone increases with the depth of intrusion, the indenter tip size affects the plastic zone volume in the shallow region of ~100 nm, resulting in the failure of the Nix–Gao model [32]. Therefore, when fitting the Nix–Gao model, we try to ensure that the interval greater than 100 nm is selected. According to the Nix–Gao model, the indentation experiment can be carried out on the unirradiated ${TiZrHfNbMo}_{0.1}$ RHEA after 1100 °C/1 h heat treatment, and the hardness $H_{unirr}$ can be obtained as the function of the indentation depth h, that is, the curve of $H_{unirr}$ and h. Then, according to Equation (2), the ${\left(H_{unirr}\right)}^{2}$ vs. 1/h curve is a straight line, as shown in Figure 5a. Its intercept with the vertical axis is $H_{0}^{2}$ and its slope is $H_{0}^{2}{\bar{h}}^{*}$ [33]. This will yield the values $H_{0}$ and ${\bar{h}}^{*}$. By substituting the values of $H_{0}$ and ${\bar{h}}^{*}$ into Equation (2) [20], the equation of the hardness variation with the depth in the unirradiated material can be obtained, and the specific fitting results are shown in Figure 5b.

(2) $$H_{unirr}=H_{0}\sqrt{1+\frac{{\bar{h}}^{*}}{h}}$$

(3) $${\bar{h}}^{*}=40.5b\alpha^{2}\mu^{2}/\left(M^{3}H_{0}^{2}tan\theta \right)$$

where $H_{unirr}$ is the hardness of the unirradiated material, μ is the shear modulus, b is the magnitude of the Burger vector, α is the dislocation-hardening coefficient, and M is the dimensionless coefficient. $H_{0}$ is the hardness value at infinite depths and is the hardness caused only by the statistical storage dislocation.

#### 3.2.2. Nanoindentation after Irradiation

During ion irradiation, defects such as dislocation loops, helium bubbles, and stacking fault tetrahedrons are generated. These irradiation-induced defects are obstacles to the dislocation motion [22,34]. Therefore, the yield strength and hardness of irradiated materials enhance. In general, the hardening of the irradiated materials depends not only on the interaction among dislocations, but also on the density and distribution of irradiation-induced defects [20].

The hardness of the material can be related to its critical decomposition shear stress through the Mises flow law [35] and the Tabor factor [36]. The spatial average values of the dislocation density before irradiation and the irradiation defect density on the indentation plastic zone are taken as ${\bar{\rho }}_{dis}\;and\;{\bar{N}}_{def}$, respectively. The average size of irradiation defects is taken as $d_{def}$, and β is taken as the hardening coefficient of irradiation defects. Therefore, the hardness of the irradiated material is given by Equation (4) [20]:

(4) $$H_{irr}=3\sqrt{3}\tau_{CRSS}^{irr}=H_{unirr}\sqrt{1+\beta^{2}{\bar{N}}_{def}d_{def}/\left(\alpha^{2}{\bar{\rho }}_{dis}\right)}$$

where $H_{unirr}=3\sqrt{3}\mu b\alpha \sqrt{{\bar{\rho }}_{dis}}$.

Then, the results of the SRIM simulation obtained by irradiation have been shown in Figure 1a. In the irradiated layer, the irradiation defect density increases with the increase in the depth, and decreases rapidly to zero after reaching the maximum irradiation depth [21,37,38]. Therefore, the spatial distribution of radiation defect density is assumed to be as follows: $N_{def}\left(x\right)=\left\{\begin{array}{c} {\left(\frac{x}{L_{d}}\right)}^{n}N_{def}^{0},\;x\leq L_{d}\\ 0,\;x>L_{d} \end{array}\right.$. In the formula, the co-ordinate x is introduced, which originates from the point on the surface of the sample that is in contact with the indentation tip at the beginning of the indentation and points to the interior of the sample being indented. $L_{d}$ defines the irradiation depth, $N_{def}^{0}$ is the peak irradiation defect density at the maximum irradiation depth, and n≥0 is the parameter describing the defect distribution profile. With the increase in irradiation dose, the defect distribution after the directional migration to the surface also has a certain influence on the parameter (n) [39]. Then, when the plastic region is still completely contained within the irradiation region, the average defect density in the plastic region is ${\bar{N}}_{def}\left(h\right)=\frac{\int_{0}^{R}\pi \left(R^{2}-x^{2}\right)N_{def}^{0}{\left(\frac{x}{L_{d}}\right)}^{n}dx}{\frac{2}{3}\pi R^{3}}=\frac{{3N}_{def}^{0}{\left(Mh\right)}^{n}}{\left(n+1\right){\left(n+3\right)L}_{d}^{n}}$. When the plastic zone exceeds the irradiation zone, the average defect density in the plastic zone is ${\bar{N}}_{def}\left(h\right)=\frac{\int_{0}^{L_{d}}\pi \left(R^{2}-x^{2}\right)N_{def}^{0}{\left(\frac{x}{L_{d}}\right)}^{n}dx}{\frac{2}{3}\pi R^{3}}=\frac{{3N}_{def}^{0}L_{d}}{2{\left(Mh\right)}^{3}}\left[\frac{{\left(Mh\right)}^{2}}{n+1}-\frac{L_{d}^{2}}{n+3}\right]$. After considering the spatial distribution of the irradiation defect density and the average dislocation density in the plastic zone, the variation of the hardness of ion-irradiated materials with the depth can be described by Equation (5) [20]:

(5) $$H_{irr}=\left\{\begin{array}{c} H_{0}\sqrt{1+\frac{{\bar{h}}^{*}}{h}+\frac{A^{2}{\bar{h}}^{*}h^{n}}{\left(n+1\right)\left(n+3\right){\left(h_{c}^{sep}\right)}^{n+1}}},\;h\leq h_{c}^{sep}\\ H_{0}\sqrt{1+\frac{{\bar{h}}^{*}}{h}+\frac{A^{2}{\bar{h}}^{*}}{2h}\left[\frac{1}{n+1}-\frac{{\left(h_{c}^{sep}\right)}^{2}}{\left(n+3\right)h^{2}}\right]},\;h>h_{c}^{sep} \end{array}\right.$$

where $A=\frac{\beta }{\alpha }M\sqrt{2btan\theta L_{d}N_{def}^{0}d_{def}}$ is the coefficient related to the ratio of the hardening coefficient α to β, the geometry of the indenter θ, the ratio coefficient M, the defect size $d_{def}$, and the irradiation damage state (represented by $N_{def}^{0}$ and $L_{d}$). $h_{c}^{sep}$ is the indentation depth at the maximum depth $L_{d}$ of the plastic zone reaching the irradiation zone. This equation can be easily simplified to the Nix–Gao model of unirradiated materials with $N_{def}^{0}=0$.

The values of $H_{0}$ and ${\bar{h}}^{*}$ are obtained by fitting the Nix–Gao model data of unirradiated materials. On this basis, the experimental $H_{irr}$ vs. h curve can be transformed into $g\left(h\right)\equiv {\left(\frac{H_{irr}}{H_{0}}\right)}^{2}-\frac{{\bar{h}}^{*}}{h}-1$ vs. h curve. According to Equation (6), it is transformed into [20]

(6) $$g\left(h\right)\equiv {\left(\frac{H_{irr}}{H_{0}}\right)}^{2}-\frac{{\bar{h}}^{*}}{h}-1=\left\{\begin{array}{c} Ph^{n},\;h\leq h_{c}^{sep}\\ Z\frac{1}{h}-Q\frac{1}{h^{3}},\;h>h_{c}^{sep} \end{array}\right.$$

where

(7) $$P=\frac{A^{2}{\bar{h}}^{*}}{\left(n+1\right)\left(n+3\right){\left(h_{c}^{sep}\right)}^{n+1}},\;Z=\frac{P}{2}\left(n+3\right){\left(h_{c}^{sep}\right)}^{n+1}\;\mathrm{and}\;Q=\frac{P}{2}\left(n+1\right){\left(h_{c}^{sep}\right)}^{n+3}$$

**Figure 6:**
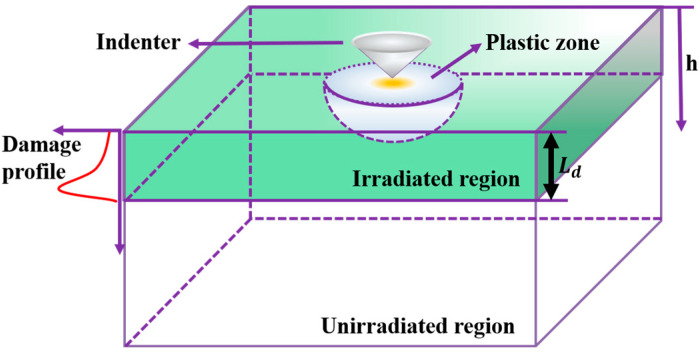
Schematic of nanoindentation of ion-irradiation metallic specimen.

Through the above formula, it reflects the idea that an irradiation layer is generated on the surface of the sample after irradiation. With the indentation of the nano-probe indentation, a plastic zone will be generated below the probe (the specific nanoindentation of the irradiated sample is shown in Figure 6). In the region $h\leq h_{c}^{sep}$, $g\left(h\right)=Ph^{n}$ increases with the increase in the indentation depth, h. In the region of $h_{c}^{sep}<h\leq h_{c}^{max}$, $g\left(h\right)=Z/h-Q/h^{3}$ firstly increases with the increase in the indentation depth h until the threshold depth $h_{c}^{max}$ is reached, and $g\left(h\right)=Z/h-Q/h^{3}$ reaches the maximum. When ${h>h}_{c}^{max}$, $g\left(h\right)=Z/h-Q/h^{3}$ decreases monotonously with the increase in the indentation depth h. The reason for the non-monotonic result is that, when $h\leq h_{c}^{sep}$, with the increase in the indentation depth h, the plastic zone expands deeper into the material with a higher defect density, so the average defect density on the plastic zone increases. When ${h>h}_{c}^{sep}$, the plastic region just touches the unirradiated region, and the plastic region expands around to include more volumes of materials with high defect density, so the average defect density of the plastic region still increases with the increasing indentation depth, h. After $h=h_{c}^{max}$, when the plastic zone below the irradiation zone is large enough, the average defect density in the plastic zone begins to decrease with the further increase in the indentation depth, h.

In addition, this threshold $h_{c}^{max}$ can be obtained by calculating the derivative of $g\left(h\right)=Z/h-Q/h^{3}$ to h and setting it to zero, that is, $d\left(Z/h-Q/h^{3}\right)/dh=0$ [20].

(8) $$h_{c}^{max}=\sqrt{\frac{3\left(n+1\right)}{n+3}}h_{c}^{sep}$$

**Figure 7:**
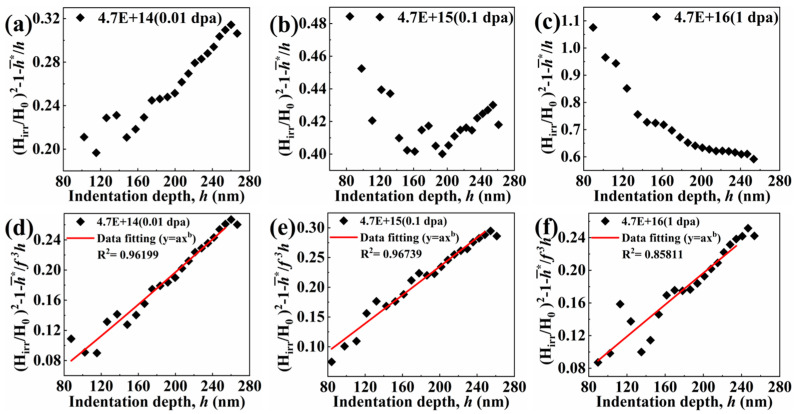
(**a**–**c**) Corresponding points of $g\left(h\right)\;versus\;h$ at different doses after heat treatment; and (**d**–**f**) corresponding points and fitting curves of $\varphi \left(h\right)\;versus\;h$ after scale factor f correction at different doses.

It is puzzling that the measured points of the samples with doses of 0.1 dpa and 1 dpa in the experimentally measured depth (30–260 nm), that is, the irradiated layer, do not conform to the trend in Equation (6) during the parameterization process. As Figure 7b,c show, $g\left(h\right)=Z/h-Q/h^{3}$ has a confusing trend, or even an opposite trend. Therefore, the reason is that the two parameters of $H_{0}$ and ${\bar{h}}^{*}$ remain unchanged before and after irradiation. They may change under the influence of irradiation, in fact. Among them, since Equation (3) shows that ${\bar{h}}^{*}$ is the characteristic length depending on the statistically stored dislocation density through $H_{0}$, which is the hardness arising from the statistically stored dislocations alone, there is a certain proportional relationship between $H_{0}$ and ${\bar{h}}^{*}$. If the trend wants to be changed in Figure 7a–c, the idea that both $H_{0}$ is unchanged and ${\bar{h}}^{*}$ is changed needs to be considered. Then, the influence factor of the ${\bar{h}}^{*}$ change is mainly the parameter, M, which describes the size of the plastic zone. $R_{s}=f\cdot R=fM\cdot h$: in the formula, the scale factor f is added to describe M, R is the radius of the plastic zone, and $R_{s}$ is the radius of the plastic zone corrected by scale factor f [40].

After introducing different scale factors in Figure 7d–f, different characteristic lengths ${\bar{h}}^{*}$ are obtained, which is to obtain the fitting curves that are more suitable for a different dpa. It can be obtained by Formula (3) [40]:

(9) $${\bar{h}}^{*}\times \left(1/f^{3}\right)=40.5b\alpha^{2}\mu^{2}/\left({\left(fM\right)}^{3}H_{0}^{2}tan\theta \right)$$

And, then, new functional relations can be constructed by Equation (6):

(10) $$\varphi \left(h\right)\equiv {\left(\frac{H_{irr}}{H_{0}}\right)}^{2}-\frac{{\bar{h}}^{*}}{f^{3}h}-1=\left\{\begin{array}{c} Ph^{n},\;h\leq h_{c}^{sep}\\ Z\frac{1}{h}-Q\frac{1}{h^{3}},\;h>h_{c}^{sep} \end{array}\right.$$

**Figure 8:**
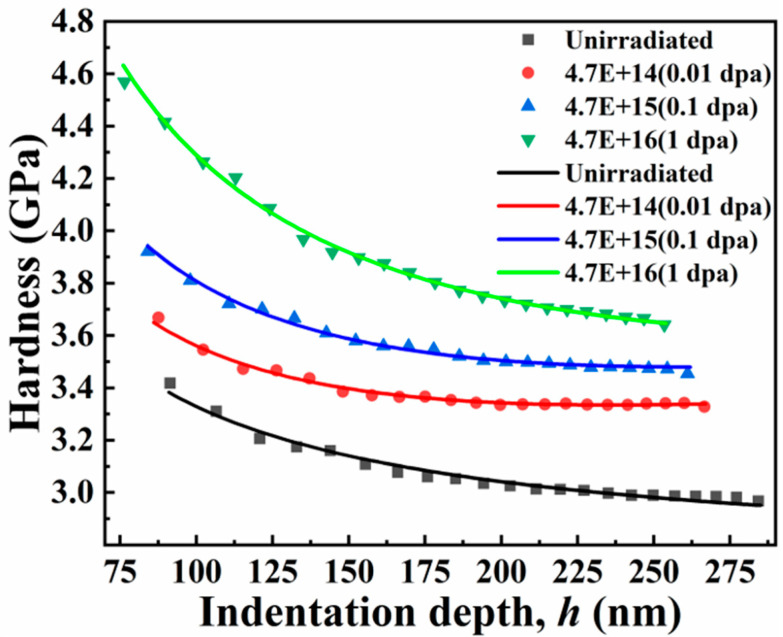
The hardness corresponding points of the alloy with the depth change after heat treatment and the curve corresponding to the scale-factor-f-modified model under unirradiated condition and different doses of irradiation.

By constantly adjusting the value of the scale factor f, it is found that the right-most hardening trend of the irradiation-hardening part in Equation (10) can be obtained within a specific scale factor range. The values of $h_{c}^{max}$, $h_{c}^{sep}$, and f of the ${TiZrHfNbMo}_{0.1}$ alloy under different doses of irradiation are shown in Table 2. It can be found from Table 2 and Figure 7d–f that the indentation depth of the nano-indentation obtained by the experiment is basically within the irradiation zone. Moreover, to minimize the impact of the surface on the nanoindentation results, the data points that measure less than 80 nm are excluded from the analysis. The hardening model with the change of depth after irradiation can well describe the size effect of indentation after irradiation considering the scale factor f. The specific fitting results are shown in Figure 8. Last but not least, the part of the elastic deformation effect is less affected and, therefore, is not considered for the time being in the selected range (80–260 nm) [41].

### 3.3. Effect of f on the Plastic Zone

**Figure 9:**
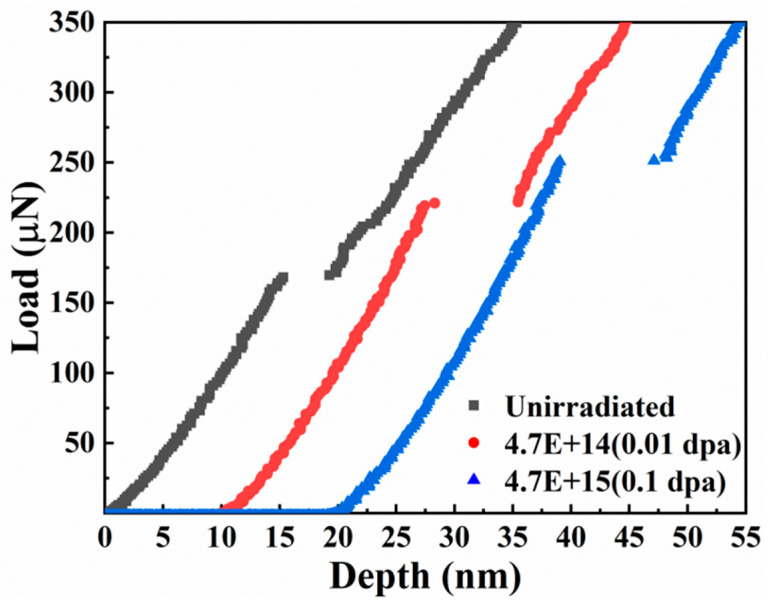
The pop-in of the alloy under unirradiated condition and different doses of irradiation after heat treatment. In order to contrast clearly, the line was translated.

In the nanoindentation of ion-irradiated samples, the defects caused by ion irradiation are distributed in the irradiation area directly below the surface. During the downward pressing of the indenter of the nanoindentation, the hemispherical plastic zone evolves [42]. The evolution of the scale factor f obtained from the parameterization in Section 3.2 can be summarized in Table 2. With the increase in irradiation dose, the scale factor f decreases continuously, so the radius of the plastic zone decreases continuously. This is consistent with the results obtained by TEM and finite element simulation in many steels and alloys [41,43,44]. For example, the normalized plastic zone size of irradiated Fe-9%Cr-ODS nanoindentation decreases in both neutron irradiation and ion irradiation [43]. In the nanoindentation of the V-4Ti alloy irradiated by hydrogen and helium ions, the maximum depth of the plastic zone decreases with the increase in damage degree [44].

f reflects the space size of the dislocation storage range, which is closely related to the nucleation and movement of dislocations. In the process of nanoindentation, the activation of dislocations is a thermal activation process of stress bias. In this process, the critical stress and the activation volume *V* are important [45,46]. As the irradiation dose increases in Figure 9, the greater the load required for pop-in to appear. It indicates that, the larger the irradiation dose, the more difficult the dislocation nucleation, and the greater the critical shear stress required for nucleation. The larger the width of pop-in, the greater the dislocation density of nucleation under the indenter [47,48]. The invisibility of pop-in under 1dpa is mainly that there are already dislocations in the material under higher irradiation damage. The original dislocations are activated to achieve plastic deformation, and the pop-in events may not be obvious. In the face of increasing nucleation dislocation density and decreasing plastic zone, it can be predicted that the activation volume of dislocations may be reduced to a certain extent.

Precisely, for the dislocation density, the statistical storage dislocation density remains unchanged. The relationship between geometrically necessary dislocation and f is shown as follows:

(11) $$\rho_{GND}=\frac{\lambda }{V}=\frac{\frac{\pi hR}{b}}{\frac{2}{3}\pi R_{s}^{3}}=\frac{3}{2}\frac{hR}{bf^{3}R^{3}}=\frac{3}{2}\frac{1}{f^{3}}\frac{{tan}^{2}\theta }{bh}$$

In the shallow layer of nanoindentation, the geometrically necessary dislocation density is dominant [40,49], and its density in Equation (11) increases with the decrease of the scale factor f. This, likewise, demonstrates that the nucleation dislocation density under the indenter raises with the increase in irradiation dose.

Furthermore, compared with pure metals, the plastic zone of RHEAs should shrink due to the lattice distortion caused by the size difference. In ion irradiation, after ion implantation into the sample, the defects such as vacancies and interstitial atoms will be produced by atomic displacement, and defect aggregation will produce dislocation loops and voids, and defects combine with helium ions to form helium bubbles. These defects will lead to serious lattice distortion in HEAs, which will inhibit the slip of dislocations and, thus, inhibit the expansion of the plastic zone. The lattice distortion caused by different types of irradiation-induced defects is different. The irradiation-induced defects formed at a larger irradiation dose cause a greater lattice distortion, so the volume of the plastic zone decreases more at a larger irradiation dose. Therefore, the scale factor f representing the size of the plastic zone can reflect the radiation resistance of the material to a certain extent. The smaller the change of the scale factor f before and after irradiation, the less of the defects produced by the material, and the better the anti-irradiation performance.

## 4. Conclusions

In this study, we mainly carried out irradiation and nanoindentation experiments on ${TiZrHfNbMo}_{0.1}$ RHEAs. The ${TiZrHfNbMo}_{0.1}$ alloy maintains a single-phase BCC structure after heat treatment. The values of $H_{0}$ and ${\bar{h}}^{*}$ can be obtained by the Nix–Gao model, so as to obtain a favorable fitting function before irradiation. After irradiation, the scale factor f is corrected by constructing the function ($\varphi \left(h\right)\equiv {\left(\frac{H_{irr}}{H_{0}}\right)}^{2}-\frac{{\bar{h}}^{*}}{f^{3}h}-1$ vs. h) in the nanoindentation. As the irradiation dose raises, the value of f becomes smaller and smaller. It reveals that the size of the plastic zone becomes smaller when the degree of the irradiation damage is greater, and the geometrically necessary dislocation density is also increasing. On the other hand, the larger irradiation damage aggravates the lattice distortion, and the more serious lattice distortion inhibits the expansion of the plastic zone. In short, the increasing number of irradiation-induced defects combined with the serious lattice distortion and slow diffusion of the high-entropy alloy result in a significant change in the size of the plastic region where the geometrically necessary dislocations are stored. Therefore, the constructed functional relationship ($\varphi \left(h\right)\equiv {\left(\frac{H_{irr}}{H_{0}}\right)}^{2}-\frac{{\bar{h}}^{*}}{f^{3}h}-1$ vs. h) is more suitable for multicomponent high-entropy alloys.

## Figures and Tables

**Table 1:** The contact depth ($h_{c}$), hardness (*H*), equivalent elastic modulus ($E_{r}$), and hardening rate of the alloy under different dpa.

Dpa	$h_{c}$ (nm)	*H* (GPa)	$E_{r}$ (GPa)	Hardening Rate (%)
0	284.29 ± 1.50	2.97 ± 0.03	49.32 ± 0.38	0
0.01	266.71 ± 2.19	3.32 ± 0.05	79.40 ± 0.68	11.8
0.1	261.09 ± 1.93	3.45 ± 0.05	70.82 ± 0.43	16.2
1	252.95 ± 3.00	3.66 ± 0.08	71.98 ± 0.82	23.2

**Table 2:** The corresponding $h_{c}^{max}$ and $h_{c}^{sep}$ of the alloy under different dpa, and the scale factor f.

Dpa	$h_{c}^{sep}$ (nm)	$h_{c}^{max}$ (nm)	f
0.01	210	260	0.928 ± 0.055
0.1	207	254	0.838 ± 0.040
1	165	247	0.709 ± 0.011

## Data Availability

The data that support the findings of this study are available upon request from the corresponding author. The data are not publicly available due to privacy.
